# Examining public support for comprehensive policy packages to tackle unhealthy food environments

**DOI:** 10.1017/S1368980024002532

**Published:** 2024-11-22

**Authors:** Simone Wahnschafft, Achim Spiller, Yasemin Boztuğ, Peter von Philipsborn, Dominic Lemken

**Affiliations:** 1 Research Training Group in Sustainable Food Systems, University of Göttingen, Heinrich-Düker-Weg 12, Göttingen 37073, Germany; 2 Department of Agricultural Economics and Rural Development, Marketing for Food and Agricultural Products, University of Göttingen, Platz der Göttinger Sieben 5, Göttingen 37073, Germany; 3 Department of Business Administration, University of Göttingen, Göttingen 37073, Germany; 4 Chair of Public Health and Health Services Research, Institute for Medical Information Processing, Biometry, and Epidemiology (IBE), LMU Munich, Germany Pettenkofer School of Public Health, Munich 81377, Germany; 5 Institute for Food and Resource Economics, University of Bonn, Nußallee 21, Bonn 53115, Germany

**Keywords:** Policy packaging, Food environments, Public support, Conjoint experiment

## Abstract

**Objective:**

This study examines public support – and its drivers – for comprehensive policy packages (i.e. bundles of coherent policy measures introduced together) aimed at improving food environments.

**Design:**

Participants completed an online survey with a choice-based conjoint experiment, where they evaluated pairs of policy packages comprising up to seven distinct food environment measures. After choosing a preferred package or opting for a single policy, participants designed their ideal policy package. Based on their choices, respondents were categorised as resistant, inclined or supportive towards policy packaging according to their frequency of opting out for single measures and the number of policies they included in their ideal package.

**Setting:**

The study was conducted in Germany via an online survey.

**Participants:**

The sample included 1200 eligible German voters, recruited based on age, gender and income quotas.

**Results:**

Based on both opt-out frequency (44·7 %) and ideal policy packaging (72·8 %) outcomes, most respondents were inclined towards policy packages. The inclusion of fiscal incentives and school-based measures in packages enhanced support, while fiscal disincentives reduced it. Key drivers of support included beliefs about the importance of diet-related issues and the role of government in regulation, while socio-demographic factors, political leaning and personal experience with diet-related disease had minimal impact.

**Conclusions:**

The results reveal public appetite for policy packages to address unhealthy food environments, contingent on package design and beliefs about the issue’s severity and legitimacy of intervention. Public health advocates should design and promote policy packages aligned with public preferences, especially given anticipated opposition from commercial interests.

Effectively and equitably addressing the global rise of unhealthy diets and the burden of chronic disease requires comprehensive public policies to improve the environments where people make daily food choices. Over the past decade, various evidence-based frameworks^(1,2)^ and international policy guidelines^(3)^ have emerged to outline essential measures for healthier food environments. While policy recommendations differ slightly, they all emphasise the need for comprehensive action. For instance, the Healthy Food Environment Policy Index (Food-EPI), a widely used framework, defines seven key domains for food environment policies: food composition, labelling, promotion, provision, retail, prices, and trade food composition, labelling, promotion, provision, retail, prices and trade^(2)^.

Despite the clarity of these recommended actions, policy uptake has been slow^(4)^. Many government strategies have focused on the provision of education and information to encourage healthier individual behaviours^(5)^, which alone overlook the environmental factors shaping dietary choices. Additionally, countries implementing food environment policies often do so on a small scale, adopting one or two isolated measures that lack the integration needed to tackle the complex drivers of unhealthy diets^(6)^.

Recently, a more comprehensive approach to improving food environments, known as policy packaging, has begun to gain traction. Policy packaging involves combining multiple policy measures designed to meet shared objectives, enhancing each measure’s effectiveness while reducing unintended consequences and improving feasibility^(7)^. This approach was first implemented by Chile in 2016 with a food environment policy package regulating the labelling, marketing and availability of ultra-processed foods and beverages, especially for children and adolescents. Several evaluations have since shown that this package significantly improved relevant public health outcomes, such as food purchasing behaviour and dietary intake^(8,9)^. Although countries in Europe have not yet adopted this approach^(10)^, guidance for the region emphasises the need for a coordinated policy ‘mix’ (i.e. package) to foster sustainable, healthy diets^(11)^.

In this paper, we examine public support for food environment policy packages in Germany, where recent national guidance has also highlighted the need for a comprehensive approach to improve food environments^(12)^. We explore public support for these policy packages for three main reasons. First, while public support is not the only factor limiting policy adoption, it has been identified as a major obstacle, alongside strong industry opposition and a lack of political leadership^(13)^. Public opinion has influenced policy outcomes in real-world cases, such as the soda ban in New York City and Denmark’s fat tax, both of which faced public backlash and industry pressure, leading to their failure^(14,15)^. Second, previous studies examining public support for food policies have focused on comparing support across single policy measures^(16,17)^, with a key message emerging that public support is often lowest for policies that are most effective and equitable in improving food choices^(18)^. However, as this comparison of policy measures against one another does not align with current policy guidance towards integrated, comprehensive policymaking, it is important to examine public support in the context of policy packages. Finally, research in other policy areas suggests that packaging policies can mitigate opposition to less popular policies by pairing them with popular ones^(19,20)^. In the food environment policy arena, increased support for sugary drinks taxes has been observed when revenues are earmarked to ‘compensate’ for the perceived ‘costs’, such as by funding programmes for disease prevention or improvement of healthcare services^(21)^, highlighting the potential of policy packages to enhance public support that we aim to expand upon in this paper.

Several aspects of policy design have been demonstrated to influence public support for policies to foster healthier food environments. One such aspect is the effect of the measure on individual choice. Here, it is useful to introduce the Nuffield Ladder of Intervention, a framework used to taxonomise public health measures based on their level of intrusiveness on individual choice, from measures that enable choice to those that restrict it^(22)^. Generally, policies that are more restrictive on individual choice tend to be more effective and equitable in their effects but face lower public support, thereby posing a challenge to the political feasibility of adopting effective policies^(18,23)^. Simplified further, measures can be characterised here as those that either ‘pull’ individuals towards desired behaviours (i.e. inform or enable choice, guide by incentive) or ‘push’ them away from undesired behaviours (i.e. restrict or eliminate choice, guide by disincentive). Accordingly, push measures tend to be less popular than pull measures^(24)^. Another influential aspect of policy design is the mechanism of action. Previous studies demonstrate that ‘fiscal’ measures, that is, taxes and subsidies, carry high visibility of policy costs and benefits relative to non-fiscal policies, or ‘behavioural’ policies, and may therefore be particularly polarising to public support^(19)^. Finally, the population that is targeted by a policy measure has also been found to modulate support, with higher support observed for those measures that target those perceived to be particularly vulnerable to unhealthy food environments, such as children and adolescents or adults of low socio-economic status^(18)^.

To examine public support for policy packages to improve the healthfulness of food environments, we take advantage of the recently conducted Food-EPI assessment in Germany. Based on input from a national, multi-sectoral expert panel, this assessment put forth a list of priority policy measures that should be adopted in the German context to improve the food environment based on anticipated population health impact, feasibility of adoption, and equity of impact^(25)^. Drawing upon a selected subset of seven priority policy measures from this assessment, we examine the following questions:

- To what extent do voters support policy packages to improve the healthfulness of food environments?
- How does the design of the policy package influence support for policy packages?
- Which characteristics of voters themselves influence support for policy packages?

## Methods

### Experimental design

We conducted a conjoint experiment embedded in an online survey, a method commonly used to assess voter preferences for public policies^(26)^. In this experiment, respondents evaluated a series of pairs of policy packages consisting of different combinations of up to seven policy measures. The selected measures were chosen to cover differences in three design features known to influence support for food environment policies: (a) the effect of the measure on individual choice, (b) the mechanism of action (fiscal *v*. behavioural) and (c) the target population (general public *v*. children and adolescents) (see Table 1). We categorised each measure’s impact on individual choice based on its place on the Nuffield Ladder of Intervention and as either a ‘push’ or ‘pull’ measure. Each policy measure was presented individually to respondents before the experiment with the description written in Table 1, which was taken from the Food-EPI assessment. We also added an estimated government cost or revenue impact to the description of each measure to help respondents consider policy trade-offs. Estimates were divided into three categories based on available data^(12)^: (a) under 500 million Euros, (b) 500 million to 1 billion Euros and (c) 1 to 10 billion Euros (see online supplementary material, Supplemental Table A1).

**Table 1.** Overview of selected policy measures for food environment policy packages and their policy design features

Policy measure	Description	Policy design features		
		Effect on individual choice	Mechanism of action	Target population
Nutrition education in schools	The government could promote high-quality nutrition education in kindergartens and schools by upgrading the corresponding content in the curricula of existing subjects and/or upgrading the teaching of home economics. *Expected government spending: 500 million – 1 billion Euros*	Inform (Pull)	Behavioural	Children and adolescents
Action plan to promote tap water consumption	The government could introduce measures to promote tap water consumption, including requiring food service establishments to provide tap water free of charge or for a small service fee, offering free tap water in workplace cafeterias and canteens, and promoting tap water consumption in schools and kindergartens. *Expected government spending: 500 million euros*	Enable (Pull)	Behavioural	General
Decrease value-added tax (VAT) on healthy foods	The government could decrease the VAT on healthy food products, such as fruits, vegetables, pulses and whole grains. *Reduced government revenue: 1–10 million Euros*	Guide by incentive (Pull)	Fiscal	General
Increase VAT on unhealthy foods	The government could increase the VAT on unhealthy food products, such as packaged foods high in sugar, salt and/or saturated fat. *Expected government revenue: 1–10 million Euros*	Guide by disincentive (Push)	Fiscal	General
Sugary drinks tax	The government could introduce a tax specifically on sugary drinks, such as sodas, cola drinks, energy drinks and iced teas. This tax would increase the price of sugary drinks, with higher price increases for drinks with higher sugar content. *Expected government revenue: 1–10 million Euros*	Guide by disincentive (Push)	Fiscal	General
Mandatory nutrition standards in kindergartens and schools	The government could introduce mandatory, publicly funded implementation of the nutrition standards of the German Nutrition Society (DGE) in schools and kindergartens. This would oblige cafeterias in schools and kindergartens to offer meals and snacks that align with national nutrition recommendations. *Expected government spending: 1–10 billion Euros*	Restrict (Push)	Behavioural	Children and adolescents
Mandatory nutrition standards in public institutions	The government could introduce mandatory implementation of the nutrition standards of the German Nutrition Society in public institutions, such as public offices, clinics, senior citizen facilities and universities. This would obligate cafeterias in public institutions to offer meals and snacks that align with national nutrition recommendations. *Expected government spending: 1–10 million Euros*	Restrict (Push)	Behavioural	General

Policy measures are organised from least to most intrusive on choice according to the Nuffield ladder.

**Fig. 1:**
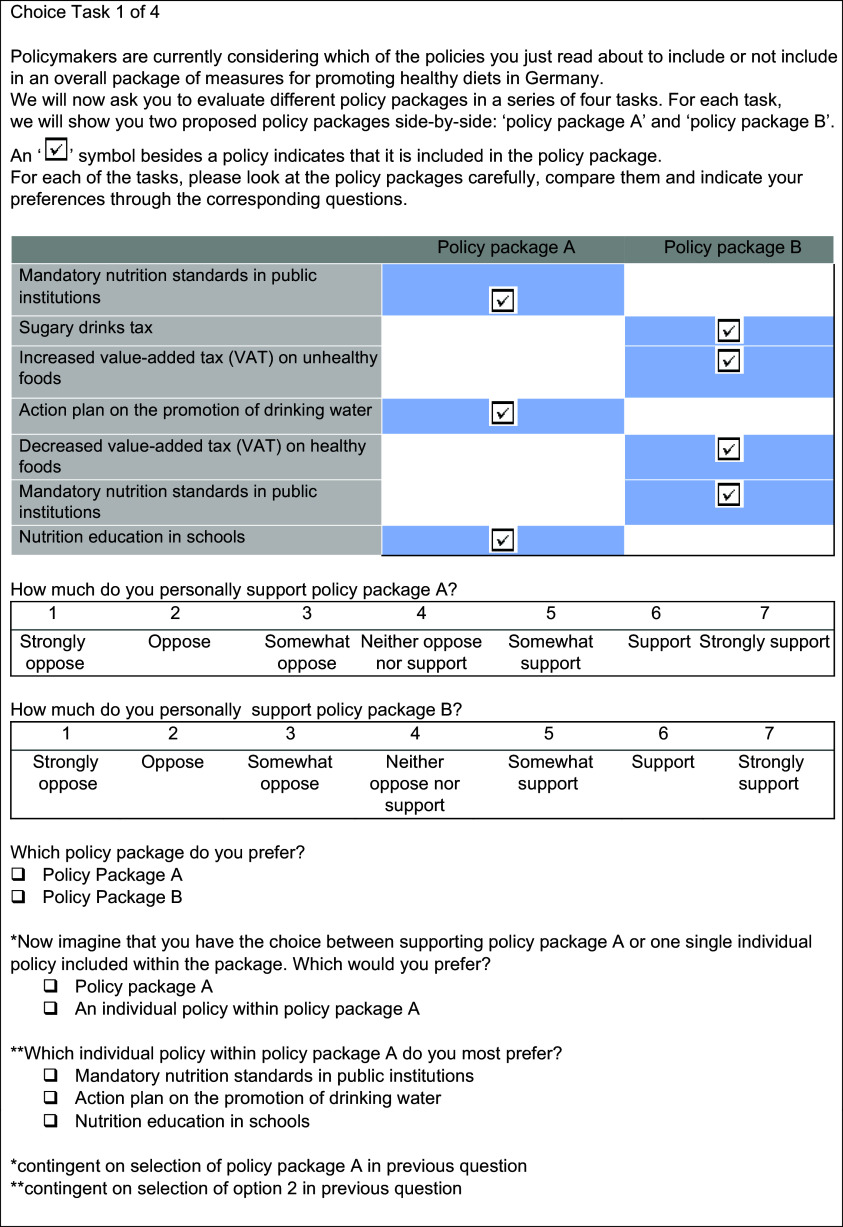
Example of paired profile design shown in each task of the conjoint experiment. Note: The order of attributes is randomised at the start and then kept constant per respondent. All questions were mandatory.

The conjoint experiment consisted of eight choice tasks, with respondents randomly divided to complete four tasks each. The conjoint experiment followed a paired profile design, in which two policy package profiles were displayed side by side in each choice task (see Fig. 1 for a sample choice task), following evidence that respondent choices in this design have been found to most closely resemble real-world voting behaviour^(27)^. In each policy package, each of the seven policy measures was either absent or present (i.e. seven attributes, with two levels each).

To avoid ordering effects, the order in which the tasks were presented was randomised. In addition, respondents were randomised to one of three versions of the choice tasks with regard to the order of the attributes (i.e. policy measures) in the package, which was then held constant throughout all choice tasks to avoid cognitive overload. The conjoint experiment was generated following a D-efficient design^(28)^ using priors calibrated based on a pre-test (*n* 94) to select choice sets that allow for an improved estimation of preference for individual policies. For the full conjoint experiment design, see online supplementary material, Supplemental Material 1 (Table A2).

Following similar conjoint experiments examining public support for packages in other policy arenas^(19,20)^, respondents first indicated their support for each of the two policy packages on a Likert scale ranging from 1 (i.e. ‘strongly oppose) to 7 (i.e. strongly support) and were subsequently prompted to indicate their preferred policy package of the two in a binary forced choice. We then included two novel approaches to examining public support for policy packages, which form the backbone of this analysis. First, upon selection of a policy package, respondents were asked whether they truly preferred the package they had selected or rather any one measure within the package, and, if the latter, were given the opportunity to opt out of the package for the single measure. Second, upon completing the conjoint experiment, respondents were asked to indicate, if given the opportunity, which of the policy measures they would include to design their own ideal policy package. Here, respondents were free to ‘drag and drop’ anywhere from zero to all seven policies into their ideal policy package.

Apart from those used to set quotas, items used to ascertain individual characteristics of respondents, such as socio-demographics and health variables, were integrated in the survey following the conjoint experiment and are described in greater detail in another section. The survey was written first in English, translated into German by a native speaker and pilot-tested amongst a heterogeneous population of German adults (*n* 18), who provided feedback on the survey structure and phrasing of questions. The pilot testing feedback was used for minor changes in question wording and ordering throughout the survey. For the full survey instrument, see online supplementary material, Supplemental Material 2. This study was provided clearance by the Ethics Committee at Georg-August-Universität Göttingen. The study has been pre-registered and is available at https://osf.io/vcsd6. The data and analysis code supporting the conclusions of this article are available via ·https://doi.org/10·7910/DVN/GJFKBX.

### Respondent selection

The survey was administered online through a market research firm to a sample representing the German voting population, with quotas set for age, gender and income based on national statistics^(29–31)^. The sample size (*n* 1200) exceeded the minimum recommended for conjoint experiments to ensure reliability^(32)^. Respondents under the age of 18 years or those who indicated ineligibility to vote in national elections were screened out, as were respondents who failed either of two attention checks that were integrated into the survey. Respondents (*n* 88) who completed the survey in less than 10 min (< 60 % of the median response time) were also excluded, as it was assumed that they did not have time to adequately process and evaluate the scenarios. A comparison of summary statistics before and after dropping these participants demonstrated negligible differences to the distribution of quota variables, indicating that these participants did not differ notably from the remainder of the participants.

### Measuring respondent characteristics

Socio-demographic variables included age, gender, income, region, parental status and political leaning. We classified regions as either former East or West Germany based on whether or not respondents resided in any of the five states that were once considered part of the German Democratic Republic (GDR) or rather in former West Germany. Political leaning was measured on a ten-point scale and grouped as far left (0–1), centre left (2–4), centre (5–6), centre right (7–8) or far right (9–10). Health status was assessed through body mass index and a binary variable indicating any diagnosis of diabetes, hypertension, CVD and/or high cholesterol. We also examined the role of beliefs about food policy, with respondents indicating their level of agreement with three statements: awareness (i.e. the high consumption of unhealthy foods and beverages causes serious problems for society), legitimacy (i.e. it is legitimate to establish collective rules for the consumption of unhealthy foods and beverages) and social norm (i.e. it is generally accepted that the consumption of unhealthy foods and beverages should be reduced). All were assessed via seven-point Likert scales, with statements drawn from a recently developed model of food policy acceptability drivers^(33)^.

### Data analysis

Support for policy packages was analysed across three outcomes. First, support ratings for each policy package on the Likert scale were collapsed into a binary variable, with a score of 5 (‘I somewhat support’) to 7 (‘I strongly support’) indicative of support. Second, we examined the frequency of opting out of policy packages in favour of single measures, recorded as a binary outcome (‘opt out’) and as an ordinal variable capturing how often respondents opted out across tasks (‘opt-out frequency’). Third, we analysed participants’ ideal policy packages, developing an ordinal ‘ideal policy package density’ variable based on the number of policy measures respondents placed in their ideal policy package. We used ‘opt-out frequency’ and ‘ideal package density’ variables to classify respondents into three groups: (1) resistant to packaging (high opt-out frequency, low ideal policy package density), (2) inclined towards packaging but sensitive to design (moderate frequency, moderate density) and (3) supportive of packaging (low frequency, high density). For a summary of the construction of the support tendency categories, based on the latter two outcomes of interest, see Table 2.

**Table 2.** Support tendencies towards policy packages for improving food environments based on A) opt-out frequency and B) ideal policy package density

Support tendency	Outcome variable	
	Opt-out frequency	Ideal policy package density
Resistant	High (opted out of 3–4 of policy packages)	Low (selected 0–1 measures in ideal policy package)
Inclined	Moderate (opted out of 1–2 policy packages)	Moderate (selected 2–5 measures in ideal policy package)
Supportive	Low (never opted out)	High (selected 6–7 measures in ideal policy package)

Next, we utilised both the conjoint experiment and ideal policy packaging exercise to examine the effect of policy package design features on support. First, we used two fixed effects logistic regression models to assess how each of the seven policy measures influenced support for packages: one model examined whether each measure’s presence or absence affected support, and the other looked at opting out^(34)^. Both models controlled for whether measures appeared in ‘package A’ or ‘package B’. The marginal effects of each measure are shown visually, with full regression results available in online supplementary material, Supplemental Material 1 (Table A3). Based on these findings, each policy measure was categorised as having a ‘positive’, ‘negative’, ‘neutral’ or ‘unclear’ effect on support, depending on statistical significance and direction of impact.

In the ideal package exercise, we descriptively analysed patterns to see which single measures or combinations of measures were commonly chosen by respondents. We also looked at how certain features of policy design – that is, the effect on individual choice, mechanism of action and target population – appeared in respondents’ preferred packages.

Finally, we assessed the influence of respondent characteristics on support using two ordinal logistic regression models based on opt-out frequency and ideal package density outcomes^(35)^. Marginal effects are displayed visually, with complete results in online supplementary material, Supplemental Materials (Table A4). Health and socio-demographic variables were standardised to compare their relevance, and multi-collinearity checks showed all variables were suitable for separate analysis^(36)^.

## Results

### Respondent characteristics

The final cleaned dataset (*n* 1112) closely resembles the general population based on quota variables, though with slight over-representation of older age groups due to the exclusion of respondents under the age of 18 years. For a summary of sample statistics and comparisons to available national statistics, see Table 3.

**Table 3.** Summary of sample statistics and comparison to available German national statistics for respondent characteristics

		Percentage	
Variable	*n*	Sample	Population*
Gender†			
Female	533	47·9	49·2
Male	574	51·6	50·8
Age‡			
18–24	89	8·00	7·00
25–34	154	13·8	13·0
35–44	165	14·8	13·0
45–54	172	15·5	13·0
55–64	222	20·0	16·0
Income (Euro, Monthly Net)			
< 1000	54	4·90	4·90
1000–1500	146	13·1	12·9
1501–2000	137	12·3	11·8
2001–2500	147	13·2	13·5
2501–5000	391	35·2	34·7
> 5000	237	21·3	22·2
Employment			
Working (full-time, part-time)	650	44·7	67·9
Unemployed (temporarily or fully)	19	1·71	3·0
Retired	326	29·3	–
Other (homemaker, disability status, student)	117	10·5	–
Regional residence§			
Former GDR	187	16·8	27·7
Former West Germany	925	83·2	72·3
Parental status of child < 18 years of age			
Yes	247	22·2	–
No	865	77·8	–
BMI			
Underweight (BMI < 18)	16	1·44	2·3
Normal weight (18 < BMI < 25)	479	43·1	44·2
Overweight (25 < BMI < 30)	392	35·3	34·5
Obese (BMI > 30)	196	17·6	19·0
Prevalence of nutrition-related disease			
Hypertension	344	30·9	31·8
Heart disease	98	8·81	12·0
Diabetes	120	10·8	7·2
High cholesterol	236	21·4	–
Political leaning			
Left (far)	86	7·73	–
Left (centre)	297	26·7	–
Centre	540	48·6	–
Right (centre)	156	14·0	–
Right (far)	33	2·97	–
Political party support¶			
CDU	107	22·1	22·5
CSU	21	4·30	6·00
SPD	108	22·3	26·4
FDP	40	8·30	8·70
Grune	113	23·4	14·0
Die Linke	32	6·61	5·00
AfD	51	10·5	10·1
Other	12	2·48	7·30

GDR, German Democratic Republic.

* Population data sources: Age^(29)^, Gender^(30)^, Income^(31)^, Employment^(30)^, Regional residence^(45)^, BMI^(46)^, Hypertension^(47)^, Heart disease^(48)^, Diabetes^(49)^, Political party support^(50)^.

† Five respondents either did not identify as male or female or elected not to report their gender.

‡ Two respondents did not report age.

§ Former GDR includes Mecklenburg-Vorpommern, Brandenburg, Sachsen, Sachsen-Anhalt and Thüringen.

¶ Includes 484 respondents (43·5 % of cleaned sample) who stated they did support a particular party in the 2021 Bundestag election. CDU = Christian Democratic Union of Germany; CSU = Christian Social Union in Bavaria; SPD = Social Democratic Party of Germany; FDP = Free Democratic Party; AfD = Alternative for Germany.

### Support for policy packages

On average, 65·4 % of respondents supported the food environment policy packages presented in the conjoint experiment. The package with the lowest support (43·0 %) included a sugary drinks tax and mandatory nutrition standards for public institutions, making it the only package with less than majority support. In contrast, the package with the highest support (81·1 %) included enhanced nutrition education in schools, a plan to promote drinking water, and mandatory nutrition standards in schools and public institutions.

Despite relatively high support indicated for policy packages, respondents opted out relatively frequently for single-policy measures – this occurred in almost half (46·6 %) of all tasks. Looking at the respondent level, just under half (44·7 %) were inclined to support policy packages, demonstrating a moderate tendency to opt out of selected policy packages to instead prefer single measures within those policy packages. Another 34·3 % were resistant to packaging, preferring single policies in nearly all choice tasks (see Table 4).

**Table 4.** Distribution of respondents’ support tendencies for food environment policy packages based on (A) opt-out frequency and (B) ideal policy package density

		B) Ideal policy package density			Total
Policy package support tendency		Supportive *High density*	Inclined *Moderate density*	Resistant *Low density*	
A) Opt-out frequency	Supportive *Low frequency*	81 7·28	138 12·4	16 1·44	235 21·1
	Inclined *Moderate frequency*	88 7·91	362 32·5	46 4·14	496 44·7
	Resistant *High frequency*	31 2·79	310 27·9	40 3·59	381 34·3
Total		200 18·0	810 72·8	102 9·17	1112 100

Number of respondents listed in the top row of each cell; percentage of total respondents listed in the bottom row.

Interestingly, resistance to policy packages decreased when respondents were allowed to design their own ideal packages: 81·4 % of those resistant to the fixed packages in the conjoint experiment were still inclined to support packaging when able to select measures themselves. Overall, the majority of participants (72·8 %) were inclined towards policy packaging here, stopping just short of including all or almost all the available measures into their ideal policy package. Notably, those supportive of combining all or nearly all measures (18·0 %) nearly doubled those resistant to packaging (9·17 %) in the ideal package task. A small subset of respondents (4·23 %) showed inconsistent preferences between the conjoint experiment and the ideal policy package task; however, most respondents were inclined towards supporting some form of food policy package, with package design appearing to play a crucial role in support.

### Effects of policy package design on support

**Fig. 2:**
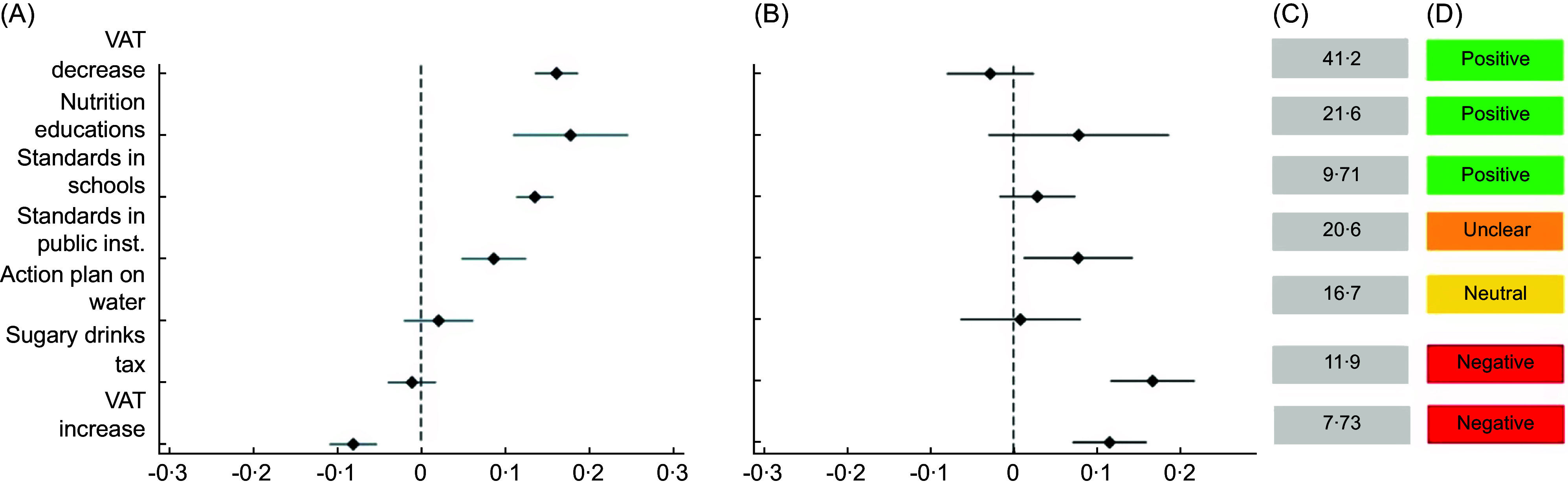
Effect of seven policy measure attributes on support for food environment policy packages, including (a) marginal effects on support for policy packages, (b) marginal effects on opting out of policy packages, (c) percentage of respondents who preferred the measure as a single measure in lieu of a package and (d) overall effect on policy package support based on (a) support and (b) opt-out outcomes. For the full regression results tables, see online supplementary material, Supplemental Material 1 (Table A3). VAT, value-added tax.

We observed both positive and negative effects of different policy measures on whether respondents (a) supported a package and/or (b) opted out in favour of a single measure (see Fig. 2). Fiscal disincentives, such as a value-added tax (VAT) increase on unhealthy foods, showed the greatest negative impact. Including this measure lowered the odds of supporting a package by 0·65 (95 % CI 0·56, 0·74) and increased the odds of opting out by 1·68 (95 % CI 1·36, 2·07). Similarly, while the sugary drinks tax did not significantly affect support for a package, it more than doubled the likelihood of opting out (OR = 2·12; 95 % CI 1·66, 2·70). These fiscal measures were only selected as single preferred policies by 7·73 % and 11·9 % of respondents, respectively, reflecting their low support for standalone adoption.

Conversely, adding a fiscal incentive, such as a VAT decrease on healthy foods, had one of the strongest positive effects on support, more than doubling the odds of respondents supporting a package (OR = 2·39; 95 CI 2·03, 2·81). This measure was also popular as a standalone policy, favoured by 41·2 % of respondents, and did not significantly increase the likelihood of opting out of a policy package, indicating it was well accepted both individually and as part of a broader package.

No behavioural policy measure had a distinctly negative impact on support. Implementing mandatory nutrition standards in public institutions showed mixed effects. While it increased the likelihood of supporting a package (OR = 1·60; 95 CI 1·30, 1·96), it also raised the odds of opting out (OR = 1·60; 95 CI 1·30, 1·96). In contrast, mandatory nutrition standards in kindergartens and schools had a clearly positive effect, more than doubling the odds of support (OR = 2·08; 95 CI 1·81, 2·40) without increasing the odds of opting out. Nutrition education in schools showed a similar positive effect (OR = 2·61; 95 CI 1·85, 3·71) and did not affect opt-out odds. An action plan to promote drinking water access demonstrated no significant effect on support.

These patterns were largely corroborated by respondents’ choices when designing their own ideal packages. Fiscal disincentives remained the least popular: only 34·5 % of respondents included the VAT increase in their ideal package, and 43·4 % included the sugary drinks tax (see Table 5). In contrast, the VAT decrease was most popular, selected by 82·3 % of respondents. Again, school-based standards were preferred over public institution standards, with 66·6 % including the former and 41·2 % the latter.

**Table 5.** Ideal package combinations of policy measures, showing the percentage of respondents including each combination in their ideal package

Policy measure (*n*)	VAT decrease	Nutrition education	Standards – schools	Standards – public	Action plan on water	Sugary drinks tax	VAT increase
VAT decrease (915)	82·3						
Nutrition education (828)	65·2*	74·5					
Standards – schools (741)	57·9*	57·2*	66·6				
Standards – public (458)	36·9	36·1	38·0	41·2			
Action plan on water (592)	48·2	43·7	39·2	27·9	53·3		
Sugary drinks tax (482)	39·3	34·3	30·9	19·6	23·8	43·4	
VAT increase (384)	32·4	27·5	24·7	16·9	20·1	29·3	34·5

VAT, value-added tax.

Boxes in grey indicate the percentage of respondents who placed a given policy measure listed in the first column (e.g. 82·3 % of respondents included a VAT decrease in their ideal policy package).

* Policy combinations selected by a majority (> 50 %) of respondents as part of an ideal policy package.

Three specific measure combinations were selected by a majority (> 50 %) of respondents as part of their ideal package. The VAT decrease was commonly paired with mandatory school standards (57·9 %) and nutrition education (65·2 %). Additionally, a combination of school standards and nutrition education focused on children was favoured by 57·2 % of respondents.

Examining the packaging of policies by design features (see Table 6), most respondents (81·5 %) preferred packages that included both push and pull measures. Few respondents preferred either only fiscal (4·23 %) or only behavioural measures (6·38 %) in their ideal package, with most favouring a mix (82·9 %). Likewise, most respondents (73·1 %) preferred a combination of policies targeting both the general population and children. Finally, similar proportions of respondents supported packages with only those policies that demonstrated a ‘positive’ effect on support in the conjoint experiment (44·6 %) as packages that also included policies that demonstrated a ‘negative’ effect on support in the conjoint experiment (48·3 %).

**Table 6.** Design preferences for ideal policy packages, by policy measure design features

Policy measure design feature			Percentage of total respondents who included measures with [A], [B] or [A] + [B] features in ideal policy package		
	[A]	[B]	[A]	[B]	[A] + [B]
Effect on individual choice	Push	Pull	0·99	11·1	81·5
Mechanism	Fiscal	Behavioural	4·23	6·38	82·9
Targeting	Children and adolescents	General population	1·89	9·62	82·0
Effect on policy package support	Negative	Positive	0·27	44·6	48·3

Results are expressed as the percentage of the total population (*n*: 1112) of respondents.

### Effects of respondent characteristics on support

**Fig. 3:**
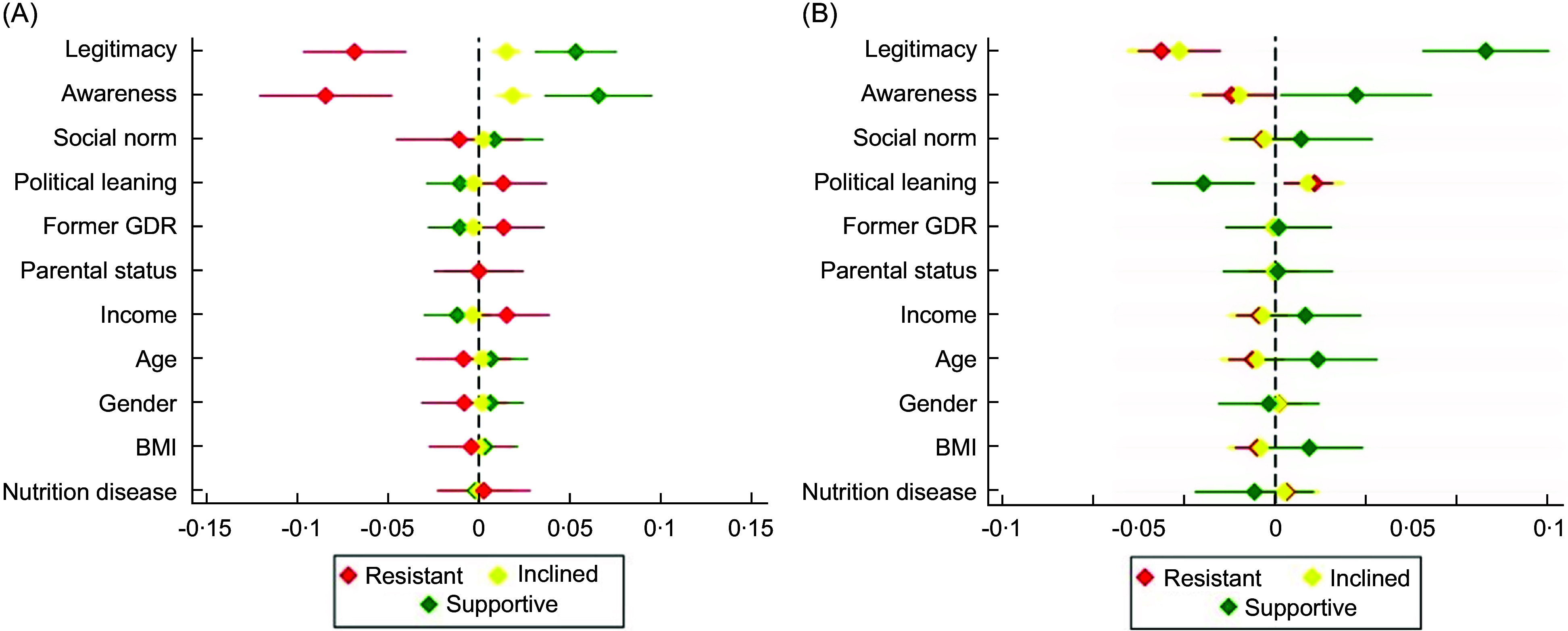
Marginal effects of respondent beliefs, political orientation, socio-demographics and health status characteristics on captured support tendency for food environment policy packaging (i.e. supportive, inclined and resistant), as reflected by (a) opt-out frequency and (b) ideal policy package density. For the full regression results table, see online supplementary material, Supplemental Material 1 (Table A4).

Respondents who (a) believed the high consumption of unhealthy food and beverages causes problems for society and (b) believed it legitimate to intervene on this consumption were significantly more likely to be supportive of policy packaging. This relationship was exhibited both in reducing the frequency with which respondents opted out of set policy packages in the conjoint experiment (OR_a_ = 0·66; 95 % CI 0·55, 0·80 | OR_b_ = 0·72; 95 % CI 0·62, 0·83) and increasing the number of policy measures selected as part of an ideal policy package (OR_a_ = 1·24; 95 % CI 1·01, 1·51 | OR_b_ = 1·75; 95 % CI 1·48, 2·05). Conversely, low accordance with these beliefs increased the odds of resistance towards policy packaging, again across both the opt-out frequency and ideal policy package density outcomes (see Fig. 3).

The effect of these beliefs was inconsistent in predicting inclination towards policy packaging across the two outcomes, as high accordance with these beliefs increased the odds of being inclined for the former and decreased them for the latter. This inconsistency suggests that these beliefs are more influential at the extremes of policy package support (i.e. resistance *v*. strong support). Meanwhile, the belief that others in society also support intervention had no significant impact on support for packaging in either outcome.

Political conservatism significantly affected support for policy packaging, but only in the designing ideal packages. Namely, conservatives were less likely to fully support packaging and more likely to be either resistant or moderately inclined towards it (OR = 0·83; 95 % CI 0·72, 0·95). Despite the high prevalence of overweight, obesity (52·9 %) and diet-related diseases (43·2 %) in the sample, these health factors did not significantly affect support for packaging. Overall, beliefs and political leaning were the strongest predictors of support for food environment policy packages, while other socio-demographic factors were largely irrelevant.

## Discussion

We demonstrate that there is an appetite amongst the public for improving food environments through comprehensive policy packages. Illustratively, all but one policy package received majority support, even though respondents were introduced to the anticipated costs of each policy measure prior to the conjoint experiment. In both their tendency to opt out of policy packages in the conjoint experiment and design their own ideal policy packages, respondents demonstrated an overall inclination to policy packages; however, they demonstrated that policy package design mattered to their support. Our results add nuance to our understanding of public support for policies aimed at healthier food environments. For example, while previous studies suggest higher support for ‘pull’ measures (those that inform, enable or incentivise healthy choices) over ‘push’ measures (those that restrict or penalise unhealthy choices), our findings suggest that when integrated policymaking is possible, preferences are more complex. Indeed, when given the chance to design their own interventions, most respondents chose combinations that included both push and pull measures, as well as both fiscal and behavioural measures, and both measures targeted at children/adolescents and at adults. In addition, although some measures showed clear positive or negative impacts on support in the conjoint experiment, a near majority of respondents still preferred ideal packages that combined both positive and negative measures, aligning with research suggesting that bundling less popular policies with popular ones can increase overall support^(19,20)^.

Our study suggests promising opportunities to create effective and publicly supported policy packages by focusing on school food environments. In Germany, mandatory standards for schools and kindergartens were rated as a high-priority policy by experts in the Food-EPI assessment due to their expected impact on health, equity and feasibility^(25)^. Our results show strong public support for these standards, particularly when combined with investments in nutrition education in schools. This is relevant in the German context, where a previous attempt to introduce a ‘meat-free day’ in workplace cafeterias met with strong public backlash^(37)^. Our findings suggest that similar standards focused on schools would likely be better received and could serve as the foundation for a broader policy package to improve food environments. Evidence from Chile shows how school-focused policies can drive positive attitudinal and behavioural changes. For example, Chilean mothers reported that schools became central to promoting healthier food behaviours, and young children even influenced their parents’ attitudes and purchasing decisions^(38)^. This recommendation could also apply in other EU countries, where improving school food environments is a key recommendation for promoting healthier food choices^(18)^.

Finally, our findings on the factors driving support for policy packages have important implications for advocacy efforts. In accordance with previous surveys conducted amongst citizens in Europe^(16)^, beliefs about the importance of nutrition policy were more influential in driving support (particularly at the extremes of support and resistance) than socio-demographic factors or personal experience with diet-related diseases. The latter, while striking given a high prevalence of diet-related disease in our sample, is not altogether unsurprising, as previous studies have been mixed in terms of reception for policy action amongst those who are particularly targeted by it^(39)^, including amongst overweight/obese individuals^(17,40)^, parents of children and/or adolescents^(21,40)^, and frequent consumers of unhealthy foods and beverages, including sugary drinks^(21,40)^ and fast food^(16)^. These findings emphasise that public support for food environment policy packages cannot be easily pinpointed along socio-demographic, chronic disease status or even political lines, but rather cuts across these delineations of voters. These results suggest that communication efforts that emphasise the role of environmental factors in shaping dietary behaviours could be key to fostering public support. This is highly relevant in contexts like Germany, where many people believe that diet-related diseases like obesity are primarily due to individual choices, such as overeating or lack of exercise, and see personal responsibility as central to a healthy diet^(41)^.

Regarding limitations, while preferable to standard survey approaches to elicit more rigorous assessments of support^(18)^, conjoint experiments are still subject to a degree of social desirability bias^(42)^. We tried to reduce this by including estimated government spending or revenue to help respondents consider trade-offs. In terms of the opt-out outcome, we were mindful of potential status quo bias, as policy packages were presented as the default option^(43)^. To address this, we compared support across both the conjoint experiment and ideal package tasks, where respondents actively chose measures. Interestingly, contrary to what would be expected based on status quo bias, we found resistance to packaging was higher when packages were the default and lower when respondents could fully customise their ideal package. However, it is important to note that this does not fully mirror real-world policymaking, where respondents may not engage with policies in such depth. In addition, we provided detailed descriptions of each policy measure, though real-world exposure to policy information is often more limited. However, we found this structure important for respondents to have the best opportunity to understand each policy measure before evaluating them in a complex policy package.

Going forward, additional research should further examine the role that individual beliefs play in underpinning support – or lack thereof – for policy packages. Namely, while this study found a significant influence of beliefs related to the issue of unhealthy diets and legitimacy to intervene, it would be important to understand which perceptions regarding policy packages such as their effectiveness, equitable impact and coherence across policy measures best predict support. In addition, recommendations for policy packages to improve food environments increasingly integrate health as a component of a broader concept of sustainability, which also encompasses social, environmental and animal welfare goals^(11,12)^, which are not included in this study and should be reflected in future studies. Finally, while our study provides valuable insights into public preferences for food environment policy packages, we recognise that our structured, experimental approach may not fully reflect the complexities of real-world public opinion. In practical settings, public support can be influenced by dynamic factors such as media framing, political partisanship and lobbying by commercial interests, which were not fully addressed in this study. Future research should explore these elements to better understand how public support may evolve in response to real-world pressures and political discourse.

Despite these limitations, our study provides foundational evidence that the public is generally supportive of policy packages to make meaningful changes in food environments. This is especially important given political inertia that stems in part from a perceived lack of public demand for action^(15)^. When effectively leveraged, as demonstrated for example in the adoption of a food environment policy package in Argentina^(44)^, public support can help mitigate imbalances of power between health and industry interests, making policy passage more achievable. Thoughtful policy design and clear communication will be essential to build strong public support for effective food environment policies.

## Supporting information

Wahnschafft et al. supplementary material 1 Wahnschafft et al. supplementary material

Wahnschafft et al. supplementary material 2 Wahnschafft et al. supplementary material

## Supplementary material

For supplementary material/s referred to in this article, please visit https://doi.org/10.1017/S1368980024002532
