# Supplementary material for: Examining public support for comprehensive policy packages to tackle unhealthy food environments
Source: Public Health Nutr. 2024 Nov 22;28(1):e7. doi: 10.1017/S1368980024002532 (PMC11736651; doi:10.1017/S1368980024002532)
Supplement: Wahnschafft et al. supplementary material 1 — Wahnschafft et al. supplementary material [file S1368980024002532sup001.docx]

**Examining public support for comprehensive policy packages to tackle unhealthy food environments**

**Table A1. Cost and/or revenue scale for policy measures and available sources.** ‘Low’ is <500 million, ‘medium’ is 500 million – 1 billion, and ‘high’ is 1-10 billion.

| **Policy instrument** | **Cost Dimension** | **Figure Available** | **Range** | **Additional explanation** |
| --- | --- | --- | --- | --- |
| Mandatory nutrition standards for schools and kindergartens | Government spending | For state-funded daycare and school meals, additional state expenditures of approx.  5.5 billion per year can be assumed for state-funded daycare and school catering^(28)^ | High | Even if this is not funding the meals entirely, it is well over 1 billion threshold for ‘high’, so even if the costs were lower, this still provides a safe estimate. |
| Increase VAT on unhealthy foods | Expected government revenue | Danish fat tax generated $216 million in new revenue in 15 months before it was repealed ^(29)^ | High | Germany’s population is over 15x that of Denmark, likely placing anticipated revenue well above the 1 billion ‘high’ threshold. |
| Decrease VAT on healthy foods | Reduced government revenue | The recommended reduction (7% to 5%) in the value-added tax on fruit and vegetables leads to a total of  revenue shortfall of around EUR 0.5 billion per year ^(28)^ | High | The estimate given of 0.5 billion is for reduction in VAT from 7% to 5% and only on fruits and vegetables. The recommended measure from the FOOD-EPI assessment for Germany is from 19% to 7% and is also on whole grains and legumes. In this case, revenue would likely exceed 1 billion. |
| Sugary drink tax | Expected government revenue | 1.89 billion euro per year ^(28)^ | High | Estimated to be above 1 billion in the context of Germany. |
| Mandatory nutrition standards for public institutions | Government spending | Not available. | High | Assumed to be similar to adopting mandatory nutrition standards in kindergartens and schools. |
| Action plan on the promotion of drinking water | Government spending | Not available. | Low | Consensus reached amongst authors that one-time spending on infrastructure for drinking water (I.e., fountains) would cost under 500 million. |
| Nutrition education in schools | Government spending | Not available. | Medium | Consensus reached amongst collaborators. |

**Table A2. Conjoint experiment design.** ‘1’ indicates policy was present in the package.

| Choice task | 1 | 1 | 2 | 2 | 3 | 3 | 4 | 4 | 5 | 5 | 6 | 6 | 7 | 7 | 8 | 8 |
| --- | --- | --- | --- | --- | --- | --- | --- | --- | --- | --- | --- | --- | --- | --- | --- | --- |
| Policy package | A | B | A | B | A | B | A | B | A | B | A | B | A | B | A | B |
| VAT dec | 1 | 0 | 0 | 1 | 0 | 1 | 1 | 0 | 0 | 1 | 0 | 1 | 1 | 0 | 0 | 0 |
| Nutr. Ed | 1 | 0 | 0 | 1 | 1 | 0 | 1 | 0 | 1 | 0 | 0 | 0 | 1 | 0 | 1 | 0 |
| Water plan | 0 | 1 | 0 | 1 | 0 | 1 | 0 | 1 | 1 | 0 | 0 | 0 | 0 | 1 | 0 | 1 |
| Stand. school | 1 | 0 | 0 | 1 | 1 | 0 | 0 | 1 | 1 | 0 | 0 | 1 | 1 | 0 | 0 | 1 |
| Stand. public | 1 | 0 | 1 | 0 | 0 | 1 | 1 | 0 | 1 | 0 | 0 | 0 | 0 | 1 | 0 | 1 |
| Sugar tax | 0 | 1 | 1 | 0 | 1 | 0 | 0 | 1 | 0 | 1 | 0 | 0 | 1 | 0 | 0 | 1 |
| VAT inc | 1 | 0 | 0 | 1 | 0 | 1 | 0 | 1 | 0 | 1 | 0 | 0 | 0 | 1 | 1 | 0 |

**Table A3. Logistic regressions of policy design attributes on (A) odds of supporting (binary) for policy package; and (B) odds of opting out of policy package.**

|  | **(A) Support for policy package** | | | |  | **(B) Opt out of policy package** | | | |
| --- | --- | --- | --- | --- | --- | --- | --- | --- | --- |
|  | **Odds Ratio** | **Standard Error** | **95% Confidence Interval** | |  | **Odds Ratio** | **Standard Error** | **95% Confidence Interval** | |
| VAT dec | 2.390^***^ | (0.197) | 2.032 | 2.810 |  | 0.882 | (0.104) | 0.699 | 1.112 |
| Nutr. Ed | 2.616 ^***^ | (0.465) | 1.847 | 3.705 |  | 1.422 | (0.352) | 0.875 | 2.309 |
| Water plan | 1.120 | (0.129) | 0.893 | 1.405 |  | 1.038 | (0.173) | 0.749 | 1.438 |
| Stand. school | 2.079^***^ | (0.148) | 1.808 | 2.391 |  | 1.137 | (0.121) | 0.922 | 1.402 |
| Stand. public | 1.597^***^ | (0.167) | 1.301 | 1.962 |  | 1.418^*^ | (0.215) | 1.053 | 1.909 |
| Sugar tax | 0.943 | (0.074) | 0.808 | 1.100 |  | 2.121^**^ | (0.263) | 1.663 | 2.704 |
| VAT inc | 0.647^***^ | (0.046) | 0.563 | 0.744 |  | 1.680^**^ | (0.180) | 1.361 | 2.074 |
| package A | 0.499^***^ | (0.104) | 0.332 | 0.753 |  | 0.330^**^ | (0.095) | 0.187 | 0.581 |
| Observations | 5920 |  |  |  |  | 2764 |  |  |  |

Exponentiated coefficients; Standard errors in parentheses

^*^ *p* < 0.05, ^**^ *p* < 0.01, ^***^ *p* < 0.001

**Table A4. Ordered logistic regressions of voter characteristics (health status, socio-demographics, political orientation, and beliefs) on (A) opt out tendency and (B) ideal policy package density.**

|  | **(A) Opt out frequency** | | | |  | **(B) Ideal policy package density** | | | |
| --- | --- | --- | --- | --- | --- | --- | --- | --- | --- |
|  | **Coef.** | **Standard Error** | **95% Confidence Interval** | |  | **Odds Ratio** | **Standard Error** | **95% Confidence Interval** | |
| Nutrition disease | 1.013 | (0.066) | 0.892 | 1.150 |  | 0.946 | (0.076) | 0.808 | 1.108 |
| BMI | 0.979 | (0.057) | 0.873 | 1.098 |  | 1.094 | (0.079) | 0.949 | 1.260 |
| Gender | 0.961 | (0.057) | 0.856 | 1.079 |  | 0.983 | (0.067) | 0.859 | 1.124 |
| Age | 0.959 | (0.063) | 0.844 | 1.090 |  | 1.118 | (0.090) | 0.955 | 1.310 |
| Income | 1.078 | (0.064) | 0.959 | 1.211 |  | 1.083 | (0.082) | 0.934 | 1.255 |
| Parental status | 0.999 | (0.062) | 0.885 | 1.129 |  | 1.007 | (0.075) | 0.869 | 1.165 |
| Former GDR | 1.069 | (0.061) | 0.956 | 1.195 |  | 1.009 | (0.073) | 0.876 | 1.161 |
| Political leaning | 1.067 | (0.064) | 0.948 | 1.202 |  | 0.826^**^ | (0.057) | 0.721 | 0.947 |
| Social norm | 0.948 | (0.083) | 0.799 | 1.125 |  | 1.070 | (0.104) | 0.885 | 1.293 |
| Awareness | 0.661^***^ | (0.063) | 0.549 | 0.796 |  | 1.238^**^ | (0.126) | 1.014 | 1.512 |
| Legitimacy | 0.715^***^ | (0.052) | 0.619 | 0.825 |  | 1.745^***^ | (0.144) | 1.484 | 2.052 |
| / |  |  |  |  |  |  |  |  |  |
| Cut1 | -4.631^***^ | 0.391 |  |  |  | 0.690 | (0.383) | -0.061 | 1.441 |
| Cut2 | -2.502^***^ | 0.369 |  |  |  | 4.972 | (0.430) | 4.129 | 5.814 |
| Observations | 1099 |  |  |  |  | 1099 |  |  |  |

Standard errors in parentheses

^*^ *p* < 0.05, ^**^ *p* < 0.01, ^***^ *p* < 0.001
