# Supplementary material for: Examining public support for comprehensive policy packages to tackle unhealthy food environments
Source: Public Health Nutr. 2024 Nov 22;28(1):e7. doi: 10.1017/S1368980024002532 (PMC11736651; doi:10.1017/S1368980024002532)
Supplement: Wahnschafft et al. supplementary material 2 — Wahnschafft et al. supplementary material [file S1368980024002532sup002.docx]

**Examining public support for comprehensive policy packages to tackle unhealthy food environments**

***Survey Instrument***

[Section 0: Declaration of Consent 1](#_Toc1331844657)

[Section 1: Introduction to the Survey 1](#_Toc1093570390)

[Section 2: Socio-demographic data (quotas) 1](#_Toc1788946165)

[Section 3: Policy Descriptions and Comprehension Tasks 1](#_Toc1590559394)

[Section 4: Choice Experiment Introduction 1](#_Toc525593854)

[Section 5: Choice Experiment 1](#_Toc1445441653)

[Section 6: Mode of Action – Topic Beliefs 1](#_Toc789294607)

[Section 7: Co-Variates 1](#_Toc361700373)

### Section 0: Declaration of Consent

Thank you for your interest in this survey!

This survey is conducted for research purposes. This study is for scientific purposes only and does not pursue any commercial or political objectives.

Participation in this study is voluntary. The answers are completely anonymous. The information you provide will not be stored or used in any way that could reveal your personal identity.

You can cancel the survey at any time by simply closing your browser. All responses received up to this point will be deleted. There are no negative

Consequences if you decide to cancel the survey.

Please note: You must be 18 years of age or older to participate in this study.

If you have any further questions, please feel free to contact us:

Simone Wahnschafft

Sustainable Food Systems RTG

Department of Agricultural Economics and Rural Development

Heinrich-Düker Weg 12

37073 Göttingen

ELECTRONIC CONSENT: Please select your choice below.

By clicking the "Agree" button, you confirm that:

• You have read the information listed above

• You voluntarily agree to participate.

• You are at least 18 years old

- Agree
- Reject *[End of Survey]*

*Timing Mark*

*Page Break*

### Section 1: Introduction to the Survey

Welcome to this survey! We are very grateful for your participation.

The aim of this study is to better understand public opinion on food policy. Our research will only produce meaningful results if you carefully read and consider each question and express your true personal opinion. Thank you for taking this into account!

The survey will take no more than 25 minutes to complete.

*Timing Mark*

*Page Break*

### Section 2: Socio-demographic data (quotas)

**Q2.1 [Gender]. What is your gender?**

- Male
- Female
- Miscellaneous
- Don't want to make a statement about it

*Timing Mark*

*Page Break*

**Q2.2 [Age] What age group do you belong to?**

- Under 18
- 18-24
- 35-44
- 45-54
- 55-64
- 65+

*Timing Mark*

*Page Break*

**Q2.3 [Income] What is your net monthly household income?**

*The household net income is calculated by subtracting from the gross household income (all income of the household from employment, from assets, from public and non-public transfer payments and from subletting) income/wage tax, church tax and solidarity surcharge as well as compulsory social security contributions.*

- Under €1,000
- €1,001 - €1,500
- 1.501 – 2.000 €
- 2.001 - 2.500 €
- 2.501 – 5.000 €
- €5,001 and above

*Timing Mark*

*Page Break*

**Q2.4 [Eligibility] If the Bundestag election were to take place today, would you be eligible to vote?**

- Yes
- Yes *[screened out]*

*Timing Mark*

*Page Break*

### Section 3: Policy Descriptions and Comprehension Tasks

**Q3.1 [Instruments]**

Seven measures are being considered by policy makers in Germany to improve the nutritional health of the population. Each of these seven measures is described in the following pages.

**Please read EACH description carefully and answer the relevant questions.**

| **Tax on sugary drinks.**  The government could introduce a tax specifically on sugary drinks, such as sodas, cola drinks, energy drinks and iced teas. This tax would increase the price of sugary drinks, with higher price increases for drinks with higher sugar content.  *Expected government revenue: 1-10 million Euros* |
| --- |

**Please indicate the extent to which you agree or disagree with the individual statements on this page for the following action:**

**Q3.2.1 [Effectiveness_Tax]** *The measure will be effective in promoting healthier diets among the population.*

- Strongly disagree
- Disagree
- I neither agree nor disagree
- Agree
- Totally agree

**Q3.2.2 [Coerciveness_Tax]** *The measure will restrict freedom of choice.*

- Strongly disagree
- Disagree
- I neither agree nor disagree
- Agree
- Totally agree

**Q3.2.3 [Fairness_Tax]** *The measure is unfair to people with low incomes.*

- Strongly disagree
- Disagree
- I neither agree nor disagree
- Agree
- Totally agree

**Q3.2.4. [Majority_Tax]** *A majority of citizens would agree to the implementation of this policy.*

- Strongly disagree
- Disagree
- I neither agree nor disagree
- Agree
- Totally agree

**Q3.2.5 [Support_Tax] To what extent do you personally support or oppose this measure?**

- Strongly oppose
- Oppose
- Slightly oppose
- Neither support nor oppose
- Slightly support
- Support
- Strongly support

*Timing Mark*

*Page Break*

| **Increase value-added tax (VAT) on unhealthy foods**  The government could increase the value-added tax (VAT) on unhealthy food products, such as packaged foods high in sugar, salt, and/or saturated fat.    *Expected government revenue: 1-10 million Euros* |
| --- |

**Please indicate the extent to which you agree or disagree with the individual statements on this page for the following action:**

**Q3.3.1 [Effectiveness_VATinc]** *The measure will contribute effectively to the promotion of healthier diets among the population.*

- Strongly disagree
- Disagree
- I neither agree nor disagree
- Agree
- Totally agree

**Q3.3.2 [Coerciveness_VATinc]** *The measure will restrict freedom of choice.*

- Strongly disagree
- Disagree
- I neither agree nor disagree
- Agree
- Totally agree

**Q3.3.3 [Fairness_VATinc]** *The measure is unfair to low-income people.*

- Strongly disagree
- Disagree
- I neither agree nor disagree
- Agree
- Totally agree

**Q3.3.4. [Majority_VATinc]** *A majority of citizens would agree to the implementation of this policy.*

- Strongly disagree
- Disagree
- I neither agree nor disagree
- Agree
- Totally agree

**Q3.3.5 [Support_VATinc] To what extent do you personally support or oppose this measure?**

- Strongly oppose
- Oppose
- Slightly oppose
- Neither support nor oppose
- Slightly support
- Support
- Strongly support

*Timing Mark*

*Page Break*

| **Decrease value-added tax (VAT) on healthy foods**  The government could decrease the value-added tax (VAT) on healthy food products, such as fruits, vegetables, pulses, and whole grains.  *Reduced government revenue: 1-10 million Euros* |
| --- |

**Please indicate the extent to which you agree or disagree with the individual statements on this page for the following action:**

**Q3.4.1 [Effectiveness_VATdec]** *The measure will effectively contribute to the promotion of healthier diets among the population.*

- Strongly disagree
- Disagree
- I neither agree nor disagree
- Agree
- Totally agree

**Q3.4.2 [Coerciveness_VATdec]** *The measure will restrict freedom of choice.*

- Strongly disagree
- Disagree
- I neither agree nor disagree
- Agree
- Totally agree

**Q3.4.3 [Fairness_VATdec]** *The measure is unfair to people with low incomes.*

- Strongly disagree
- Disagree
- I neither agree nor disagree
- Agree
- Totally agree

**Q3.4.4. [Majority_VATdec]** *A majority of citizens would agree to the implementation of this policy.*

- Strongly disagree
- Disagree
- I neither agree nor disagree
- Agree
- Totally agree

**Q3.4.5 [Support_VATdec] To what extent do you personally support or oppose this measure?**

- Strongly oppose
- Oppose
- Slightly oppose
- Neither support nor oppose
- Slightly support
- Support
- Strongly support

*Timing Mark*

*Page Break*

| **Nutrition education in schools.**  The government could promote high quality nutrition education in kindergartens and schools by upgrading the corresponding content in the curricula of existing subjects and/or upgrading the teaching of home economics.  *Expected government spending: 500 million – 1 billion Euros* |
| --- |

**Please indicate the extent to which you agree or disagree with the individual statements on this page for the following action:**

**Q3.5.1 [Effectiveness_NutEd]** *The measure will effectively contribute to the promotion of healthier diets in the population.*

- Strongly disagree
- Disagree
- I neither agree nor disagree
- Agree
- Totally agree

**Q3.5.2 [Coerciveness_NutEd]** *The measure will restrict freedom of choice.*

- Strongly disagree
- Disagree
- I neither agree nor disagree
- Agree
- Totally agree

**Q3.5.3 [Fairness_NutEd]** *The measure is unfair to low-income people.*

- Strongly disagree
- Disagree
- I neither agree nor disagree
- Agree
- Totally agree

**Q3.5.4. [Majority_NutEd]** *A majority of citizens would agree to the implementation of this policy.*

- Strongly disagree
- Disagree
- I neither agree nor disagree
- Agree
- Totally agree

**Q3.5.5 [Support_NutEd] To what extent do you personally support or oppose this measure?**

- Strongly oppose
- Oppose
- Slightly oppose
- Neither support nor oppose
- Slightly support
- Support
- Strongly support

*Timing Mark*

*Page Break*

| **Mandatory nutrition standards in kindergartens and schools**  The government could introduce mandatory, publicly funded implementation of the nutrition standards of the German Nutrition Society (DGE) in schools and kindergartens. This would oblige cafeterias in schools and kindergartens to offer meals and snacks that align with national nutrition recommendations.    *Government spending: 500 million euros* |
| --- |

**Please indicate the extent to which you agree or disagree with the individual statements on this page for the following action:**

**Q3.6.1 [Effectiveness_K&S]** *The measure will be effective in promoting healthier diets among the population.*

- Strongly disagree
- Disagree
- I neither agree nor disagree
- Agree
- Totally agree

**Q3.6.2 [Coerciveness_K&S]** *The measure will restrict freedom of choice.*

- Strongly disagree
- Disagree
- I neither agree nor disagree
- Agree
- Totally agree

**Q3.6.3 [Fairness_K&S]** *The measure is unfair to low-income people.*

- Strongly disagree
- Disagree
- I neither agree nor disagree
- Agree
- Totally agree

**Q3.6.4. [Majority_K&S]** *A majority of citizens would agree to the implementation of this policy.*

- Strongly disagree
- Disagree
- I neither agree nor disagree
- Agree
- Totally agree

**Q3.6.5 [Support_K&S] To what extent do you personally support or oppose this measure?**

- Strongly oppose
- Oppose
- Slightly oppose
- Neither support nor oppose
- Slightly support
- Support
- Strongly support

*Timing Mark*

*Page Break*

| **Mandatory nutritional standards for other public institutions.**  The government could introduce mandatory implementation of the nutrition standards of the German Nutrition Society in public institutions, such as public offices, clinics, senior citizen facilities and universities. This would obligate cafeterias in public institutions to offer meals and snacks that align with national nutrition recommendations.    *Expected government spending: 1-10 million Euros* |
| --- |

**Please indicate the extent to which you agree or disagree with the individual statements on this page for the following action:**

**Q3.7.1 [Effectiveness_Public]** *The measure will contribute effectively to the promotion of healthier diets in the population.*

- Strongly disagree
- Disagree
- I neither agree nor disagree
- Agree
- Totally agree

**Q3.7.2 [Coerciveness_Public]** *The measure will restrict freedom of choice.*

- Strongly disagree
- Disagree
- I neither agree nor disagree
- Agree
- Totally agree

**Q3.7.3 [Fairness_Public]** *The measure is unfair to low-income people.*

- Strongly disagree
- Disagree
- I neither agree nor disagree
- Agree
- Totally agree

**Q3.7.4. [Majority_Public]** *A majority of citizens would agree to the implementation of this policy.*

- Strongly disagree
- Disagree
- I neither agree nor disagree
- Agree
- Totally agree

**Q3.7.5 [Support_Public] To what extent do you personally support or oppose this measure?**

- Strongly oppose
- Oppose
- Slightly oppose
- Neither support nor oppose
- Slightly support
- Support
- Strongly support

*Timing Mark*

*Page Break*

| **Action plan to promote tap water consumption.**  The government could introduce measures to promote tap water consumption, including requiring food service establishments to provide tap water free of charge or for a small service fee, offering free tap water in workplace cafeterias and canteens, and promoting tap water consumption in schools and kindergartens.    *Expected government spending: 500 million euros* |
| --- |

**Please indicate the extent to which you agree or disagree with the individual statements on this page for the following action:**

**Q3.8.1 [Effectiveness_Water]** *The measure will contribute effectively to the promotion of healthier diets among the population.*

- Strongly disagree
- Disagree
- I neither agree nor disagree
- Agree
- Totally agree

**Q3.8.2 [Coerciveness_Water]** *The measure will limit freedom of choice.*

- Strongly disagree
- Disagree
- I neither agree nor disagree
- Agree
- Totally agree

**Q3.8.3 [Fairness_Water]** *The measure is unfair to people with low incomes.*

- Strongly disagree
- Disagree
- I neither agree nor disagree
- Agree
- Totally agree

**Q3.8.4. [Majority_Water]** *A majority of citizens would agree to the implementation of this policy.*

- Strongly disagree
- Disagree
- I neither agree nor disagree
- Agree
- Totally agree

**Q3.8.5 [Support_Water] To what extent do you personally support or oppose this measure?**

- Strongly oppose
- Oppose
- Slightly oppose
- Neither support nor oppose
- Slightly support
- Support
- Strongly support

**Q3.9 [Attention_screen]** Which of the following measures did you read NOTHING about in this section?

- Tax on sugary drinks
- Reformulation of Salt Content in Packaged Foods
- Nutrition education in schools

*Timing Mark*

*Page Break*

### Section 4: Choice Experiment Introduction

**Q4.1 [Choice_Experiment]**

Policymakers are currently considering which of the measures you just read about should be included in an overall package to promote healthy eating in Germany and which should not.

We will now ask you to evaluate different sets of measures in a series of five tasks. For each task, we will show you two proposed sets of measures side by side: "Package A" and "Package B". A "
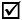
" next to an action indicates that it is included in the package.

For each of the five tasks, please carefully look at the packages of measures, compare them and answer the corresponding questions.

*Timing Mark*

*Page Break*

### Section 5: Choice Experiment

Note: This is an example of a selection task. Each participant will answer a series of 9 choices, including the follow-up questions listed here.

**Q5.1 [CT1.1]**

|  | Policy package A | Policy package B |
| --- | --- | --- |
| **Increase in Value Added Tax (VAT) on unhealthy foods** |  | 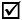 |
| **Reduction of Value Added Tax (VAT) on healthy food** |  |  |
| **Tax on sugary drinks** | 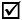 |  |
| **Binding quality standards for daycare and school catering.** | 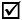 |  |
| **Regulating the marketing of unhealthy foods to children** | 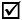 |  |
| **Mandatory nutritional standards for other public institutions.** |  | 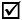 |
| **Action plan to promote tap water consumption** | 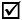 |  |
| **School Fruit & Vegetable Program** |  | 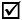 |
| **Nutrition education in schools.** |  | 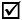 |

**Q5.2 [CT1.2]** To what extent do you personally support policy package A?

- Strongly oppose
- Oppose
- Slightly oppose
- Neither support nor oppose
- Slightly support
- Support
- Strongly support

**Q5.3 [CT1.3]** To what extent do you personally support policy package B?

- Strongly oppose
- Oppose
- Slightly oppose
- Neither support nor oppose
- Slightly support
- Support
- Strongly support

**Q5.4 [CT1.4]** Which policy package do you prefer?

- Policy package A
- Policy package B

**Q5.5 [CT1.5]** Now imagine that you had the choice between policy package [A/B] or one single individual policy included within the package. What would you prefer?

- Policy package [A/B]
- An individual policy within policy package [A/B]

**Q5.6 [CT1.6]** Which individual measure within package [A/B] do you most prefer? [Depending on the answer to the question "A single measure within the package of measures [A/B]" in the previous question]

- Tax on sugary drinks
- Binding quality standards for daycare and school catering.
- Action plan to promote tap water consumption
- Nutrition education in schools.
- Mandatory nutritional standards for other public institutions.
- Increase in VAT on unhealthy food
- Reduction of VAT on healthy food

**Q5.7 [Attention_screen_2]** What consumer behaviour is being sought to change by the policies described above?

- Eating habits
- Energy consumption in households
- Use of the car

**Q5.8 [Ideal_Package]** You have completed the section on the evaluation of the packages of measures.

Now imagine that you could put together your ideal package of measures from the seven measures that politicians in Germany are currently considering.

Please indicate which measures you would include in your ideal package of measures by dragging the measures into the box below.

Note: You can select as many or as few actions for the package as you want. The order in which you place the policy measures in the ideal package does not matter.

- Sugary drinks tax
- Mandatory nutrition standards in kindergartens and schools
- Mandatory nutrition standards in other public institutions
- Action plan to promote tap water consumption
- Nutrition education in schools.
- Increase VAT on unhealthy food
- Decrease VAT on healthy food

Your ideal package of measures

|  |
| --- |

### Section 6: Mode of Action – Topic Beliefs

**For each of the following statements, please indicate the extent to which you agree or disagree.**

**Q7.1 [Awareness]** *The high consumption of unhealthy foods and beverages causes serious problems for society.*

- Strongly disagree
- Disagree
- I neither agree nor disagree
- Agree
- Totally agree

**Q7.2 [Legitimacy]** *It is legitimate to establish collective rules for the consumption of unhealthy foods and beverages*

- Strongly disagree
- Disagree
- I neither agree nor disagree
- Agree
- Totally agree

**Q7.3 [Social_Norm]** *It is generally accepted that the consumption of unhealthy foods and beverages should be reduced*

- Strongly disagree
- Disagree
- I neither agree nor disagree
- Agree
- Totally agree

### Section 7: Co-Variates

**Q8.1 [Height]** How tall are you (in centimeters)?

_ _ [ *Open box]*

*Timing Mark*

*Page Break*

**Q8.2 [Employment]** Which statement best describes your employment status?

- Full-time employment
- Part-time employment
- Temporarily Exempt
- Unemployed
- Pensioner
- Housewife/husband
- Permanently unable to work
- Student
- Other

*Timing Mark*

*Page Break*

**Q8.3 [Weight]** How much do you weigh (in kilograms)?

_ _ [ *Open box]*

*Timing Mark*

*Page Break*

**Q8.4 [Region]** Please select the state in which you currently reside:

- Baden-Württemberg
- Bavaria
- Berlin
- Brandenburg
- Bremen
- Hamburg
- Hesse
- Mecklenburg-Western Pomerania
- Lower Saxony
- North Rhine-Westphalia
- Rhineland-Palatinate
- Saarland
- Saxony
- Saxony-Anhalt
- Schleswig-Holstein
- Thuringia

*Timing Mark*

*Page Break*

**Q8.5 [Parental_Status]** Are you the parent of a child under the age of 18?

- Yes
- No

*Timing Mark*

*Page Break*

**Q8.6 [Nutrition_related disease]** Have you ever been told by a doctor that you have any of the following diseases?

|  | Yes | No | I don't want to say |
| --- | --- | --- | --- |
| Hypertension |  |  |  |
| High cholesterol |  |  |  |
| Heart disease |  |  |  |
| Diabetes |  |  |  |

*Timing Mark*

*Page Break*

**Q8.7 [Political_leaning]** Political issues are referred to as "left" and "right". How would you rate your own views on a scale from left (1) to right (10)?

*Timing Mark*

*Page Break*

**Q8.8 [Party_affiliation]** Do you consider yourself a supporter of a particular political party, or is there one party you feel closer to than another?

- Yes
- No
- I don't know

*Timing Mark*

*Page Break*

**Q8.9 [Party_identification]** Which party is this?

- CDU - Christian Democratic Union of Germany
- CSU – Christian Social Union in Bavaria
- SPD – Social Democratic Party of Germany
- FDP – Free Democratic Party
- Greens - Alliance 90 / The Greens
- - The Linke – Linkspartei
- AfD – Alternative for Germany
- Other party (please specify) *[Open]*
